# Polysaccharide Based Scaffolds for Soft Tissue Engineering Applications

**DOI:** 10.3390/polym11010001

**Published:** 2018-12-20

**Authors:** Sanjay Tiwari, Rahul Patil, Pratap Bahadur

**Affiliations:** 1 Maliba Pharmacy College, UKA Tarsadia University, Gopal-Vidyanagar Campus, Surat 394350, Gujarat, India; tiwarisanju@gmail.com (S.T.); rahul.patil2294@gmail.com (R.P.); 2 Chemistry Department, Veer Narmad South Gujarat University, Surat 395007, Gujarat, India

**Keywords:** Polysaccharides, scaffolds, bioresorbable materials, cell adhesion, soft tissues, regeneration, chemical modification

## Abstract

Soft tissue reconstructs require materials that form three-dimensional (3-D) structures supportive to cell proliferation and regenerative processes. Polysaccharides, due to their hydrophilicity, biocompatibility, biodegradability, abundance, and presence of derivatizable functional groups, are distinctive scaffold materials. Superior mechanical properties, physiological signaling, and tunable tissue response have been achieved through chemical modification of polysaccharides. Moreover, an appropriate formulation strategy enables spatial placement of the scaffold to a targeted site. With the advent of newer technologies, these preparations can be tailor-made for responding to alterations in temperature, pH, or other physiological stimuli. In this review, we discuss the developmental and biological aspects of scaffolds prepared from four polysaccharides, viz. alginic acid (ALG), chitosan (CHI), hyaluronic acid (HA), and dextran (DEX). Clinical studies on these scaffolds are also discussed.

## 1. Introduction

Soft tissues are complex fiber-reinforced structures, generally distinguishable from hard tissues by their high water content [1]. They are continuously invaded by trauma, invasive surgery, and aging. This often leads to impaired physiological functions, large scale tissue loss, and even organ failure [2]. The restorative approaches include direct administration of primary or genetically engineered cells of auto-, allo-, or xenogeneic origin [3,4], and transplantation of cells seeded into tissue-like three dimensional (3D) scaffolds. Devoid of a stiff matrix, the former approach is associated with serious obstacles, such as the rapid escape of cells, suboptimal dispersion, insufficient vascularisation, donor site morbidity, potent immunogenic response, and long-term administration of immunosuppressive agents [5,6,7,8,9,10]. The implantation of autologous cells is challenging due to difficulty in harvesting clinical-grade cells in sufficient number, especially in aged recipients or when the damage is high [11]. Moreover, cell harvesting requires a second surgical site. This two-stage procedure increases surgery time and patients may suffer nerve damage at the harvest site [12]. The instillation procedure via traditional hand held injections imposes a pronounced surgical stress on suspended cells [13]. Studies report that 80%–90% of transplanted cells die within the first 72 h of injection [14]. More importantly, cellular de-differentiation during in vitro propagation may alter the biosynthetic properties of autologous cells [15].

Seeding the lineage- and tissue-specific progenitors, derived from patient’s normal tissue or donor, into scaffolds is a rapidly expanding tissue engineering (TE) alternative. In the 1980s, TE was understood as the application of prosthetic devices and surgical manipulation of tissues [16]. Evolution TE as a modern science is dedicated to the experiments of Vacanti et al., who developed the first tissue engineered scaffold to be used in a human [17]. An advanced understanding on TE as an interdisciplinary science emerged from one of the most cited articles published in Science by Langer and Vacanti [18].

Unlike cell suspension, seeded scaffolds exhibit a more predictable transport of high density cells into the defect site [14,19,20,21,22,23,24,25,26]. Therefore, it is essential that the structure and composition of scaffolds emulate the complexity of target tissue and mimic the confluent extracellular matrix (ECM). This is accomplished through inclusion of synergistic cell types, ECM proteins, and angiogenic factors into the scaffold [27,28]. ECM proteins and growth factors facilitate cooperative signaling and thus, augment the regenerative response [29]. Researchers have identified key peptide sequences (*viz.* RGD, YIGSR, and REDV) among large non-cellular binding domains of ECM proteins. Functionalization of scaffold material with these short peptides has shown to significantly enhance the cell attachment and proliferation. Of note, short peptides can conveniently be synthesized with desirable functionalities and be attached to polymer end groups while conserving the native properties of ECM protein [30,31,32,33]. Local placement of scaffolds, loaded with tissue-specific regenerative components, concentrates the payload in the region of interest and stimulates regeneration through defined biomolecular recognition events. Occasionally, acellular scaffold is administered to the recipient wherein regeneration leans over recruitment of native cells into the implant. This strategy has been successfully tested by Stevens et al. [34] for neobone formation. Authors have demonstrated the generation of mineralized compact bone exhibiting the expression of histological markers and mechanical properties of native bone.

It is desirable that the scaffold is stably located and offers a hospitable environment for tissue regeneration. In accordance, its performance is evaluated by the matrix stability, biocompatibility, and achievement of tissue-specific physiological signaling (Table 1). Mechanical properties are evaluated in terms of storage and loss moduli, and gel strength. These properties can be modulated either by changing the crosslinking density and molecular weight of the polymer, or through incorporation of additional components [35,36]. Nevertheless, ensuing characteristics must be appropriate for manipulation during implantation [14]. Porosity and pore interconnectivity facilitate the metabolic exchange, waste disposal, colonization, and survival of entrapped cells [37,38]. Biocompatibility with seeded cells and host tissues has been studied using validated assay procedures. Material safety has been evaluated in animal models to verify that unseeded scaffold does not induce cell infiltration or aberrant histological changes in the neighboring tissues [39]. Finally, the scaffold should provide molecular signals for driving complex multi-cellular processes and get degraded in concert with cells proliferation [40,41]. The by-products of material degradation must not induce local or systemic adverse events [42,43]. 

## 2. Scaffolds Developed from Polysaccharides

**Figure 1:**
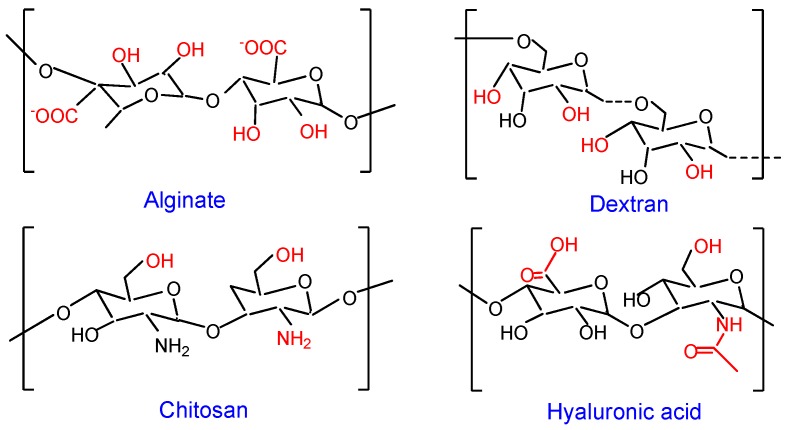
Schematic structure of polysaccharides. Active centre in the repeating unit of each polysaccharide is shown in red font.

A variety of macromolecules, ranging from synthetic to natural polymers, have been explored for the fabrication of scaffolds. Despite flexible material properties, synthetic polymers find limited TE applications as they lack biological cues inherent in many natural polymers [19,30]. Biomacromolecules (polysaccharides and proteins), derived from both animal and plants, are receiving wider interest in scaffold development [44,45]. Indeed, scaffold research has advanced in recent years due to polysaccharides, such as chitosan [46,47], alginate [43,48], dextran [49,50,51], and hyaluronic acid [41,52]. They readily form loose viscoelastic gel in aqueous vehicles via non-covalent interactions. Other attractive features include low cost, ease of derivatization, biocompatibility, and biodegradability (Figure 1). They present distinct similarity to the ECM, which is rich in glycosaminoglycans, glycoproteins, and glycolipids. The ability of polysaccharides to generate biological cues has been linked to their glycan units [53]. Cell-selective interaction has further been improved in recent years through advances in purification techniques and backbone modification [48,54,55,56,57]. Moreover, the high charge density of some polysaccharides enables the development of scaffolds using straight forward electrostatic interactions [52].

Despite these merits, the application of natural polysaccharides for scaffold preparation is associated with certain limitations. Their molecular weight distribution, branching, and sequence may not be consistent. In addition to influencing rheology, these variations may be detrimental to biorecognition events. Another noteworthy obstacle is the inferior mechanical strength of polysaccharide gels. It leads to quick hydrolysis and displacement of the formulation away from the injection site. For instance, deterioration in the viscoelasticity of hyaluronic acid occurs through the production of oligosaccharides and low molecular weight fragments [52]. Loss of viscoelasticity at physiological temperatures can be circumvented through age-old crosslinking methods [58,59]. Besides, gel strength has been improved through the incorporation of additives and/or polymers of a desirable molecular weight [60,61,62,63]. Gelatin is a good choice as a blend component (Table 2). With the presence of arginine, glycine, and aspartic acid (RGD) tripeptide in the backbone, gelatin acts as a fibroblast-attractant. Simultaneously, it promotes epithelialization and granulation tissue formation [64,65,66,67]. It has been shown to undergo proteolytic degradation without producing antigenic fragments [38]. 

In this review, we discuss the developmental and biological aspects of scaffolds prepared from four polysaccharides, viz. alginic acid (ALG), chitosan (CHI), hyaluronic acid (HA), and dextran (DEX). An emphasis is placed on scaffolds developed through physical/chemical modifications using crosslinking, grafting, polyion complexation, and blending (Table 2). Clinical studies on these scaffolds are also covered (Table 3).

### 2.1. Chitosan

Chitosan (CHI) is obtained from the partial or full deacetylation of chitin (the second most abundant biopolymer after cellulose, found in the exoskeleton of crustaceans and endoskeleton of molluscs). The protonation of amine groups during dissolution imparts a positive charge, following which it quickly adheres to negatively charged substrate surfaces. Readers are referred to some earlier reviews on biochemistry and biomedical applications of CHI [78,79]. Depending on the source, chitin exists in α- or β- crystallographic forms. As against to anti-parallel chain organization of α-form, β-chitin exhibits parallel organization. The latter configuration though allows limited probability of intermolecular hydrogen bonds, but it improves accessibility to chemical modification or deacetylation [80,81]. This is evident in the findings of Reys et al. [82], who investigated the influence of freezing temperatures (−80 and −196 °C) upon scaffold formation behavior of α- and β-chitin, with a deacetylation degree (DD) of 76.6% and 91.2%, respectively. Although both the scaffolds exhibited stability against lysozyme up to 4 weeks, those prepared at −196 °C displayed a compact structure and smaller pores. The *β*-chitin scaffold presented similar morphological features and swelling profile, but superior mechanical properties attributable to its higher DD [82].

Tissue engineering applications of CHI emerge from the properties, such as hydrophilicity, polyelectrolyte behavior, mucoadhesion, hemostatic action, and structural similarity to native extracellular proteoglycans. Its polar groups and physicochemical properties provide a favorable non-protein environment for cell adhesion and proliferation [83]. It easily forms a blend with other polymers through electrostatic interactions and confers antimicrobial properties to the final composition [1,21,84].

Mechanical properties of CHI hydrogels can be modulated through a variety of crosslinking approaches. A common approach is photo-crosslinking, achieved through the derivatization with photoactive methacrylate [85,86,87], azido [88,89], or maleic [68,90,91] groups. Such hybrid scaffolds can be conveniently produced via freeze-drying. Physical properties (rheology, absorbing capacity, morphology, crystallinity, and compressive modulus) can be tailored by controlling the degree of substitution. Studies have shown that CHI scaffolds support the attachment and proliferation of fibroblasts [21,22], chondrocytes [68], hepatocytes [69], and nerve cells [20].

### 2.2. Alginic Acid

Alginic acid or alginate (ALG) is a biocompatible and non-immunogenic polysaccharide obtained from kelp, brown algae, and some bacteria [14]. It is composed of two alternating blocks, *α*-l-guluronic acid (G) and *β*-D-mannuronic acid (M), linked via *α*-(1–4) and *β*-(1–4) glycosidic bond, respectively (Figure 1). Methods have evolved to obtain high purification grade ALG at a low cost, with negligible traces of contaminants, such as polyphenols and endotoxins [92,93]. Stable hydrogels can be developed in mild conditions by adding divalent metal cations (Ca^2+^, Sr^2+^, and Ba^2+^) to aqueous ALG solution [94,95]. Its sol-gel transition is ascribed to the formation of an “egg-box” structure upon selective binding of cations to G-blocks; a phenomenon which explains the higher elastic modulus for ALG gels richer in G blocks [96]. 

**Figure 2:**
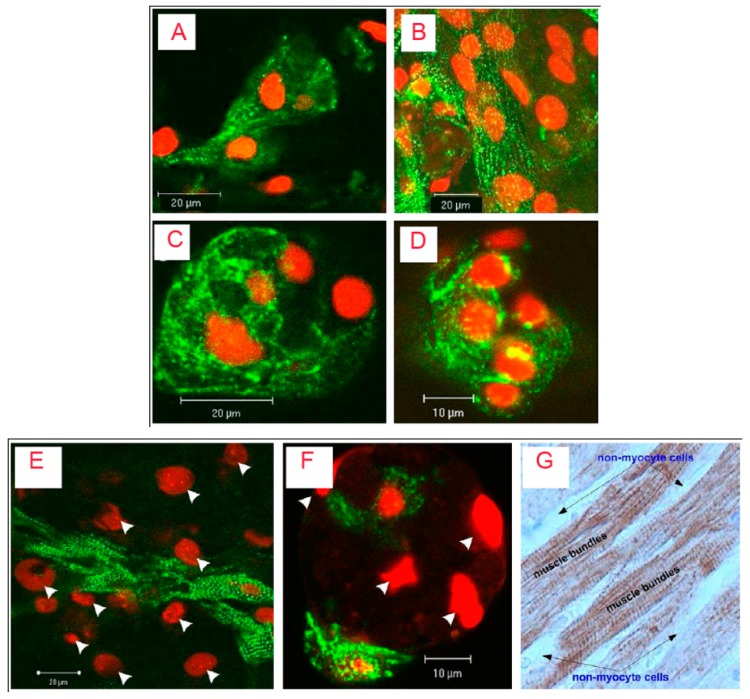
Confocal microscopy and immune histochemistry images of cardiac cells cultivated in RGD-immobilized (**A**,**B**) and unmodified ALG scaffolds (**C**,**D**) for 6 (**A**,**C**) and 12 (**B**,**D**) days. The constructs were immune stained for a-actinin (green) and nuclei (red-propidium iodide). Adjacent cardiomyocytes joined to form striated myofibers (Figure 2A, day 6), an occurrence that increased in frequency as cultivation proceeded (Figure 2B, day 12). In contrast, cardiomyocytes cultivated within the unmodified ALG scaffolds revealed unorganized myofibrils; there were fewer interactions between adjacent cardiomyocytes and myofibers were not detected (Figure 2C and D, days 6 and 12, respectively). The lower panel shows relative locations of cardiomyocytes and nonmyocyte cells (NMCs) in (**E**) RGD-immobilized and (**F**) unmodified ALG scaffold; (**G**) the native adult cardiac tissue. In E and F, only cardiomyocytes were stained for α-actinin (**green**), while all cell nuclei were stained with propidium iodide (Red). Arrow heads denote cell nuclei of NMCs. Twelve-day constructs were fixed, fluorescently stained, and examined using confocal microscopy. In G, native adult cardiac tissue was stained for troponin-T (**brown**). The NMCs surrounding cardiomyocyte bundles were negatively stained. Adult rat ventricles were paraffin-fixed, cross-sectioned, and immunostained for troponin-T. Reproduced and modified with permission from Elsevier (2011) [98].

Despite these merits, ALG is not a preferred biomaterial as it lacks cell binding motif and, therefore, exhibits poor cell adherence [97]. This has been demonstrated through a comparison between scaffolds developed from RGD-immobilized and unmodified ALG. Immobilization of the peptide promoted cell adherence to the matrix, prevented cell apoptosis, and accelerated cardiac tissue regeneration. The cardiomyocytes reorganized their myofibrils and reconstructed myofibers within six days (Figure 2). These effects were well reflected in the expression levels of α-actinin, N-cadherin and connexin-43 in cells cultured within RGD-seeded scaffolds [98]. Enhanced cell adherence upon the attachment of RGD is explained as follows. Cellular integrins link the intracellular skeleton with ECM via the RGD peptide. It initiates the cascade for cell survival and proliferation [31]. A similar argument is applicable tothe incorporation of bone-forming peptides (derived from bone morphogenetic protein-7) into scaffolds for driving osteogenesis and osteo-differentiation [99,100].

Incorporation of poly ε-caprolactone (PCL) [101,102], CHI [103], halloysite nanotubes [104], and carbon nanotubes (CNTs) [19] has been investigated to tune the mechanical properties, bioactivity, and proliferation rate of surface cells. Herein, the specific blending ratio of components eliminates the possibility of phase separation.

Acellular macroporous ALG scaffolds have shown to promote the stabilization of hepatocytes, both in vitro [105,106] and in vivo [107]. Shteyer et al. [107] demonstrated that ALG scaffolds, without implanted cells, significantly improved the survival rate of partially hepatectomized mice (87%). The animal manifested normal and prolonged aspartate- and alanine aminotransferase serum levels as compared to 2- to 20-fold increase in control groups (non-treated and collagen-treated mice). The authors correlated these findings to the non-adhesive and macroporous structure of the ALG matrix. Macroporosity enabled rapid confinement of cells within the remnant liver and caused a pronounced increase of cell polarity. Together with complimentary secretion of ECM components, growth factors, and chemokines, it created a specialized niche favorable to differentiation of remnant cells as functional hepatocytes [107]. The formation of scaffolds is dependent on pH, ion concentration, and ALG composition. Destruction of the gel network and un-controlled degradation may occur in biological buffers containing chelators or monovalent electrolytes [104,108,109]. However, scaffolds developed from covalently cross-linked ALG have shown a shape memory effect, an exploitable property while contemplating the repair of damaged annulus fibrous tissues. The formulation supported cell penetration, proliferation, and ECM deposition when cultured in intervertebral disc-like niche (low oxygen and glucose level) [110].

### 2.3. Dextran

**Figure 3:**
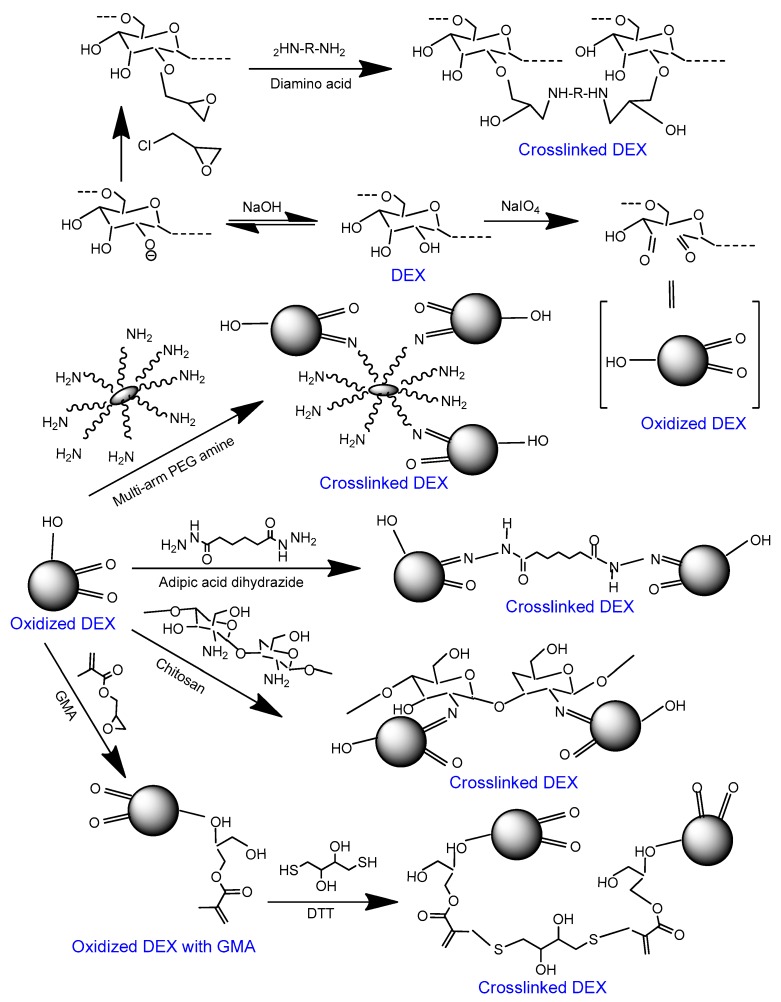
Scheme showing the crosslinking approaches for oxidized DEX. DEX can be oxidized via periodate treatment. Oxidized DEX can be crosslinked through the attachment mono-, bi-, and multi-armed amines [111,121,122,123]. Alternatively, glycidyl methacrylate (GMA) can be attached to oxidized DEX and the latter can be crosslinked with dithiothreitol (DTT) [120].

Dextran (DEX) is a bacterially-derived uncharged, linear polysaccharide composed of *α*-1,6 linked D-glucopyranose residues with a few percent of *α*-1,2-, *α*-1,3-, or *α*-1,4-linked side chains [49]. It is available in a wide range of molecular weights and undergoes enzymatic degradation in the spleen, liver, and colon [111]. Crosslinked DEX hydrogel beads have been used for aslong as low protein-binding matrices in column chromatography [112] and in microcarrier cell culture technology [113,114]. Soft tissue-engineering applications of DEX stem from its resistance to protein adsorption and cell adhesion [115]. Porous DEX hydrogels can be prepared through crosslinking mediated by hydroxyl groups present on *α*-1,6-linked d-glucose residues [116]. The polymer has three hydroxyl groups in each repeat unit, and the reactivity of these groups follows the order of C_2_>C_4_>C_3_ [117]. Several chemical modifications have been explored, yielding DEX derivatives with tailored physicochemical and functional characteristics [118,119,120] (Figure 3).

Some groups have investigated surface grafting and co-polymerization as a tool of improving the cell-adhesion of DEX [24,124,125]. Levesque et al. [125] developed scaffolds of methacrylated-DEX copolymerized with aminoethyl methacrylate. Herein, primary amine groups served as handles to covalently link RGD peptide. The adhesion and neurite outgrowth of primary embryonic chick dorsal root ganglia increased upon copolymerization. A further improvement was noticed upon peptide immobilization. Notably, direct coupling between peptide (amine) and hemiacetals of oxidized DEX destructed the conformation of peptides. At the same time, the presence of amine-pendants in the side-chain of constituent amino acids impaired the scaffold-cell interactions [124]. This has been minimized through the development of sulfhydryl-terminated peptides [125].

A recent study of Noel et al. [126] questioned the cell-selective response of ECM peptides using DEX scaffolds. Investigators illustrated the role of four ECM peptides (RGD, YIGSR, REDV, and CAG) upon adhesion and proliferation of HUVEC and AoSMC cells. A library of vinylsulfone-modified DEX was tethered with the peptides. RGD (Arg-Gly-Asp), YIGSR (Tyr-Ile-Gly-Ser-Arg), and SGIYR (Gly-Ile-Tyr-Arg) were able to enhance both HUVEC and AoSMC adhesion (showing no selectively for HUVEC over AoSMC), whereas REDV (Arg-Glu-Asp-Val) and CAG (Cys-Ala-Gly) failed in improving the cell adhesion. Interestingly, co-immobilization of vascular endothelial growth factor and RGD resulted in selective proliferation of HUVEC cells. It thus highlighted the scope of changing the conformation, sequence tuning, and lengthening of peptides as tactics to impart a cell selective response in the scaffold.

### 2.4. Hyaluronic Acid

Commercial hyaluronic acid (HA) is extracted from rooster combs, but it has also been produced using genetically engineered bacteria. Highly pure HA is available in a range of molecular weights at relatively low costs. HA and its derivatives are widely used in the cosmetics industry, medicine, and surgery. Physiochemical and biological properties, methods of modification, and drug delivery applications of HA have been described in other comprehensive reviews [52,127,128]. Its biological activity is molecular weight-dependent [129]; high molecular weight HA has anti-inflammatory and anti-angiogenic properties, whereas low-molecular weight HA possesses pro-inflammatory and pro-angiogenic activities [130,131,132]. Besides, studies show that HA promotes macrophage differentiation into the M2 phenotype [133]. Improved cellular proliferation and tissue regeneration have been demonstrated by blending with biodegradable materials [134,135,136] and coating the scaffolds with HA [137,138] and non-covalent binding [139]. These events are most likely mediated through selective interaction of HA with cell surface receptors, such as CD44, ICAM-1, and RAHMM [52,140].

**Figure 4:**
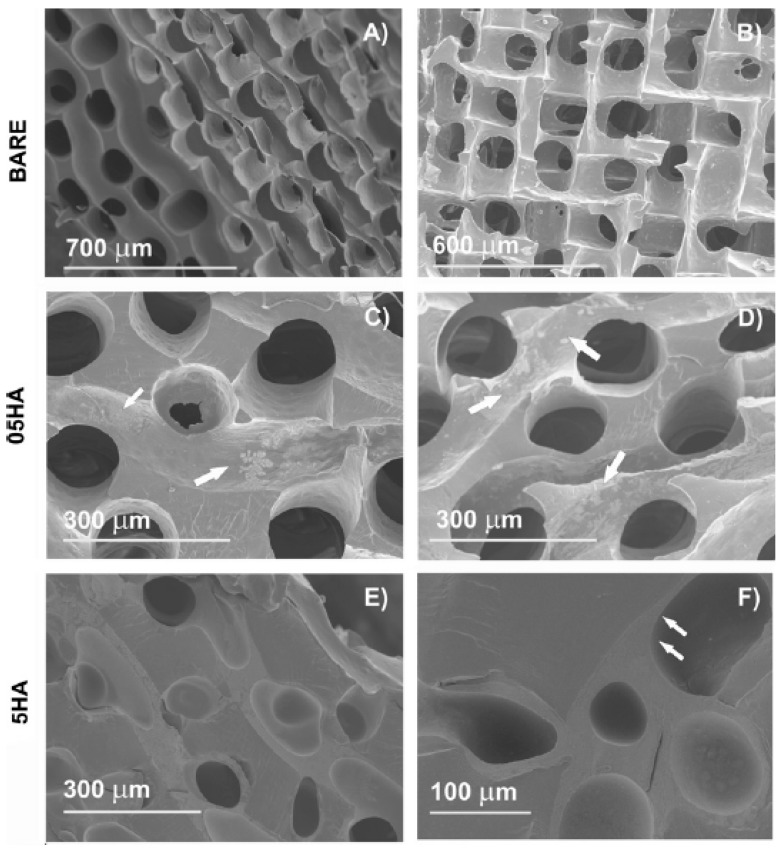
Scanning electron microscopic images of the dry poly(ethyl acrylate)(PEA) scaffolds: (**A** and **B**) Bare scaffold, cross section, and frontal view, respectively; (**C**) 05HA1; (**D**) 05HA5; (**E**) 5HA2; (**F**) 5HA5. The arrowheads point at the adsorbed HA. With one coating cycle, 0.5 wt% HA solution produced aggregates on the pore surface (shown in **C**). These aggregates become more distinct as the number of cycles increases, but a uniform layer is not obtained with 0.5wt% even after five cycles (**D**). In contrast, coating with 5wt% HA produces a uniform continuous layer after the first coating cycle. The effect of further cycles is to achieve the layer thickness. This is accompanied with a decreased pore diameter and the clogging of some pores (**E**). After the fifth cycle (**F**), the channels are filled with HA to a high degree. (05HA# and 5HA# designate the scaffolds coated, respectively, with 0.5wt% and 5wt% HA solutions, # being the number of cycles). Reproduced and modified with permission from Elsevier (2011) [138].

Kudryavtseva et al. [141] explored the effect of surface immobilized high molecular weight HA upon survival of primary human monocyte-derived macrophages. The immobilization on polylactic acid scaffolds was accomplished through atmospheric pressure cold plasma treatment. HA attachment enhanced the biocompatibility of the scaffold and stimulated its pro-angiogenic action. Interestingly, dip coating of HA (1 wt% solution) has been shown to enrich the attachment of MCF7 cells onto poly(lactic acid-co-glycolic acid) (PLGA) scaffolds [137]. Depending on the process parameters, deposited HA may have configurations ranging from thin disconnected aggregates to a thick continuous layer on the pore surface (Figure 4). Besides, layer topography may affect the swelling of scaffold and may be of interest in applications wherein resistance to normal stress is desirable [138]. For other specific applications, hybrid nanofibres can be used as reinforcement alternative [142].

While the majority of investigations have focused on exploiting the direct biological effects of HA, its incorporation intriguingly improved the mechanical strength of scaffolds and may, therefore, inhibit the cell-induced contractions. Davidenko et al. [143] investigated the influence of increasing the amount of HA upon mechanical characteristics of collagen scaffolds. Together with supporting the proliferation of 3T3-L1 preadipocytes, HA created additional crosslinks. Consequently, the scaffold exhibited improved resistance to compression and in vitro dissolution.

## 3. Approaches of Scaffold Preparation

Prototype scaffold preparations include three key components: Support material, cells, and angiogenic factors. Typically, a blend of biopolymers is employed with the objectives, such as enhancing mechanical properties, and tuning the porosity, loading property, swelling ratio, and degradation kinetics of the scaffold. Cells and growth factors either adhere to the scaffold surface [144] or get encapsulated within the matrix [145]. Formulations include hydrogel [5,91], fiber [142], film [69], and de-cellularized matrices [146,147,148]. Electrospun microfiber bundles are suturable and often exhibit an elastic modulus identical to that of native tissue [9]. Transplantation can be rendered less aggressive by developing in situ gelling formulations, which later acquire the configurations of damaged tissue [23,149]. Besides, self-crosslinking has been achieved in neat polysaccharide systems via thiolation [25].

The preparation method must be selected on the criteria, such as a desired scale of operation, controllability of steps, and batch-to-batch consistency. A general approach includes dissolving the component(s) into an aqueous vehicle and subsequent processing via solvent casting, lyophilization, electrospinning, or cryo-gelation. The weight ratio of components is adjusted to attain a desired dispersibility [57]. This is essential with the consideration that cross-linkage between the constituents may sometimes offset the hydrophilicity and pore size of polysaccharide scaffolds [74,150].

**Figure 5:**
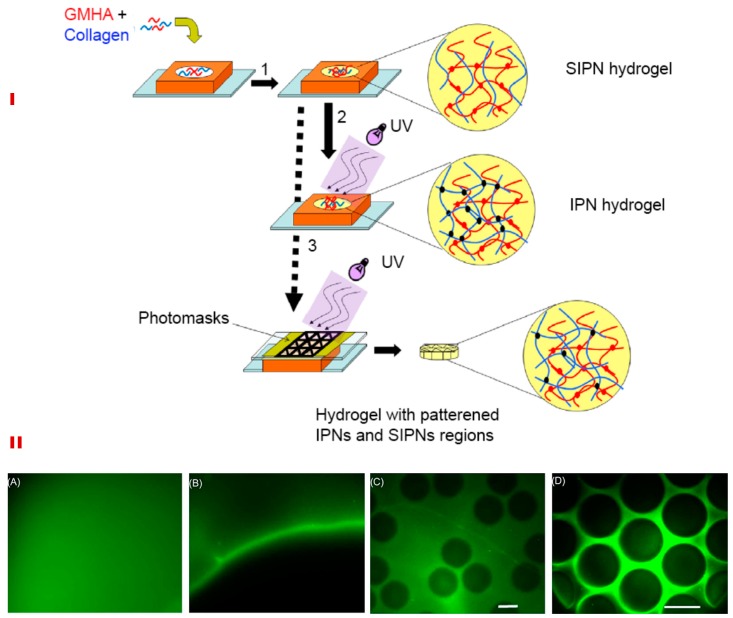
Schematic for synthesizing IPNs, SIPNs, and photopatterned hydrogels (**I**). HA and collagen solution were suspended in the silicone mold and collagen was permitted to undergo fibrillogenesis at 37°C (1). This resulted in the formation of a SIPN, which was then exposed to ultra-violet light to yield a full IPN (2). Alternatively, photo patterning was performed using a photomask, which resulted in SIPN and IPN patterns within a single hydrogel (3) (**I**). The lower panel (**II**) shows the macro- and micro-patterned hydrogels formed due to differential crosslinking densities. A macropatterned hydrogel is shown in which half was exposed to UV before (**A**) and after washing the un-crosslinked fluorescein acrylate (**B**). In addition, (**B**) shows the interface between the macropatterned halves. Micropatterning within a single bulk hydrogel of a 500 μm thickness is shown in **C** and **D**. (Scale bar-150 μm). Reproduced and modified with permission from Elsevier (2009) [151].

The scaffold can be macro- or micro-patterned at a high accuracy using controlled chemical manipulations to achieve desirable biophysical characteristics [151]. Photo-crosslinkable interpenetrating (IPNs) and semi-interpenetrating networks (SIPNs) between COL and HA have been shown to control the structural and biomechanical properties (Figure 5I). In contrast to IPN composed of two un-crosslinked polymers exhibiting full interpenetration, SIPN consists of one crosslinked polymer entangled in another un-crosslinked polymer and hence, is mechanically inferior [52]. Such entangled networks retain the structural properties of component polymers while reinforcing the scaffold. Scaffolds developed from the IPN-SIPN blend are anisotropic; showing region-specific distribution of crosslinking density, viscoelasticity, water content, and porosity [151,152] (Figure 5II).

**Figure 6:**
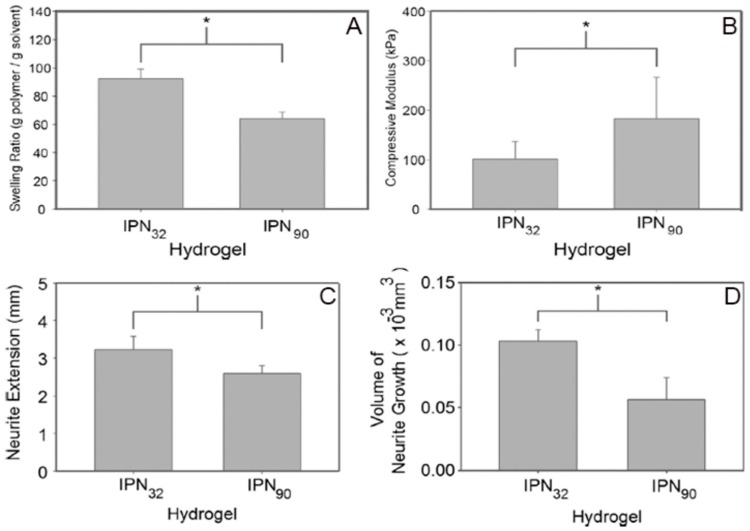
A significant increase (*p*<0.05) in the swelling ratio can be noticed in IPN_32_, with a smaller crosslinking density (**A**). Besides, a higher crosslinking density (IPN_90_) led to a significant increase (*p*<0.05) in the compressive moduli (**B**). Comparative analysis of the length of neurite extension in IPN90 and IPN32 constructs is shown in (**C**) and (**D**). A less stiff substrate allowed a longer growth, with some neurites extending up to 3.3 mm after 7 days (**C**). Analysis of the amount of neurite growth (average of five longest neurites) demonstrated that the more compliant substrate allowed superior overall growth (**D**). Reproduced and modified with permission from Elsevier (2015) [153].

Khoshakhlaghet al. [153] illustrated the effects of micro-patterning upon neurite growth using a dual hydrogel, incorporating methacrylated HA and Puramatrix (PM, a self-assembling peptide scaffold). Initially, IPN hydrogels were formulated using self-assembly of PM and photo-crosslinking of HA. It was then surrounded by photo-crosslinkable polyethylene glycol (PEG). Integration between the two compartments of hydrogel was mediated by the IPN. Crosslinkable substrates were exposed to UV radiations in geometries relevant to cover the entire gel thickness, thereby creating a desirable micro-patterning. A range of mechanical properties could be achieved by controlling the degree of methacrylation. Regions with a lesser degree of methacrylation (greater porosity) displayed better neurite outgrowth [153] (Figure 6).

Techniques, such as embossing, micro-contact printing, and layer-by-layer assembly of planer sheets, have been employed for the fabrication of micro-patterned scaffolds [154,155]. Scaffolds with honeycomb, square, and rectangle patterns (needed for specialized applications) are obtainable using these methods [156,157]. For instance, it is desirable from the cardiac scaffolds to offer electrical cues, in addition to biomimicking mechanical and topographical features. Liu et al. [158] fabricated micro-patterned cardiac patches using a tri-culture system, composed of cardiomyocytes in combination with cardiac fibroblasts and endothelial cells.

3D printing technology is also gaining popularity for its high speed and continuous scaffold design. Typically, a bioink containing cells, growth factors, and other biological solutions is printed over acellular scaffolds. A highly customized architecture can be achieved with the help of a motion-controlled multinozzle deposition system [19]. The process employs a low pressure extrusion and is operated at room temperature, with benign processing requirements. Low pressure extrusion with a large diameter nozzle helps in minimizing mechanical stress to the cells. It is, however, important that the material is sufficiently viscous to be dispensed as free standing filaments exhibiting desirable mechanical strength. The reader is referred to earlier reviews on the application of 3D printing technology in tissue engineering [159,160,161].

## 4. Clinical Status of Polysaccharide Scaffolds

The evaluation of scaffolds in a clinical set-up is necessary to validate its efficacy. Clinical reports on polysaccharide scaffolds are interesting, but the power of those findings is limited due to a small sample size, lack of a randomized control group for comparison, and the unavailability of long-term studies [162,163]. With a limited sample size and smaller follow-up period, the investigators may miss infrequent adverse events. In this landscape, the regenerative response with the test approach remains obscure. On the other hand, long-term data, acquired in a broader population, provide important indications if early risks associated with the intervention can be offset by future benefits [26,163].

Stillaert et al. [164] investigated HA-based preadipocyte-seeded scaffolds for adipo-conductive potential and efficacy in humans. Autologous cells, isolated from lipoaspirate material and seeded on HA scaffolds, were implanted subcutaneously. The scaffold displayed superior cellularity and progressive tissue integration within eight weeks of implantation. It, however, lacked angiogenic penetration since the cells were located more than 100 μm away from the native micro vasculature; beyond the diffusive capacity of oxygen [164]. The adherence of the scaffold to the lesion can be monitored by analyzing the polysaccharide content in biopsy samples [165].

Other clinical studies have employed esterified HA (HYAFF^®^). Esterification of carboxyl groups involved the preparation of a quaternary HA salt and its subsequent reaction with an esterifying agent (aliphatic, alicyclic, or aromatic alcohol) in an aprotic solvent [166]. Scaffolds based on benzyl ester (HYAFF^®^11) have been widely tested for cartilage repair (Table 2). The treatment minimizes pain and counteracts the development of arthritis [167]. It is agreed that the autologous cell-based repair technique results in the generation of hyaline-like repair tissue. It shows a lower probability of failure in comparison to fibrous repair tissue [168]. This might be the reason for the greater clinical acceptability of scaffold-based cell seeding over bone-marrow stimulating techniques for cartilage repair [165,167].

**Figure 7:**
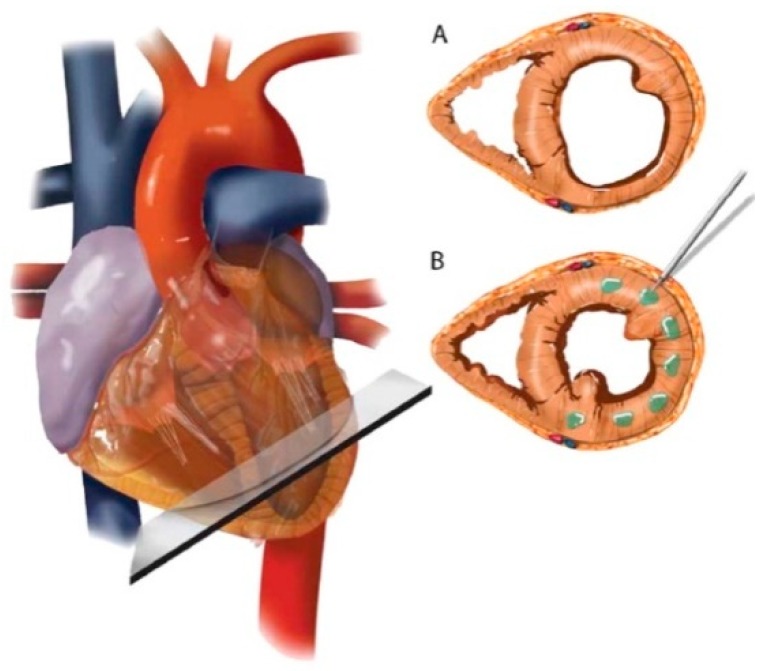
Schematic of Algisyl-LVR™ injection in the left ventricle. (**A**) Short-axis view of the mid-ventricle, half way between the apex and base. (**B**) Algisyl-LVR™ is injected at 10 to 15 locations at the mid-ventricle free wall (excluding the septum). A left thoracotomy is performed to expose the heart and the pericardium. The total number of injections for an individual patient depended on the size of the heart. Injections were separated by ≈0.5–1 cm and made at the mid-wall depth of myocardium. Reproduced with permission from Elsevier (2013) [162].

ALG has been clinically tested for improving cardiac function using Algisyl-LVR™ [162] and IK-5001 technologies [163]. Lee et al. [162] employed a proprietary gel, which transformed to a scaffold upon placement in the affected region. The formulation consisted of: (**a**) ALG component as 4.6% aqueous mannitol, and (**b**) Ca^2+^-ALG component as insoluble particles suspended in 4.6% aqueous mannitol. These solutions were extemporaneously mixed in one syringe prior to intramyocardial administration [162] (Figure 7). On the contrary, IK-5001 comprises of 1wt% ALG containing 0.3% calcium gluconate and undergoes in situ crosslinking. Its intracoronary delivery is relatively simple and does not require a unique device or complex imaging system. When injected, the formulation selectively permeates to the infracted myocardial tissue and reversibly crosslinks to form a temporary bioabsorbable cardiac scaffold in a Ca^2+^dependent manner. The scaffold then replaces the damaged ECM, reduces myocardial wall thinning and strain, and ultimately attenuates infarct expansion [163,172]. Herein, the selectivity of scaffold deposition is ascribed to abnormal microvascular permeability and elevated extracellular Ca^2+^ concentrations within the infarct zone [173].

Altogether, clinical applications of polymeric scaffolds are challenging due to intricacies of replicating the complex tissue environment without eliciting undesirable immunologic events. Tissue remodeling is often constrained by limited diffusion of oxygen and growth factors in polymeric scaffolds [174]. This has been demonstrated with reference to vascular restorative therapy in cardiac tissues. Herein, the bioresorbable nature of scaffold stimulated positive blood vessel wall remodeling and restoration of contractile functions. With these considerations, polymeric scaffolds appear superior to metallic stents. The latter often lead to distorted vessel physiology, incomplete endothelialization, and stent fracture [175,176]. At the same time, we cannot overlook the fact that behavior of polymeric materials may not be identical under dry and submerged conditions. It is, therefore, suggestive to preliminarily map the localized changes in structural integrity vis-a-vis macroscopic performance of scaffolds under practical use conditions [177]. This can certainly minimize the clinical failure of polymeric scaffolds.

## 5. Conclusions and Perspective

Preservation of local organ function via tissue regeneration is a definitive component of post-operative care. Regenerative treatment leans over the development of advanced biomaterials, processing thereof as 3D scaffolds, and investigating the manner in which scaffold material cooperates with the seeded cells, proteins, and growth factors in order to augment the natural repair mechanisms. Polysaccharide based materials have shown endless promise for developing the tissue scaffolds. Ongoing advancements in polysaccharide chemistry and nanotechnology have enabled the integration of mechanical, topographical, and biological cues into these materials for stably recapitulating the tissue-scale organization. Importantly, these recreated structures may act as customized tissue surrogates for the screening of new drug molecules [178].

While debating the translational application of scaffolds, it is of interest to look forward to upcoming technologies for mass production and setting quality control parameters. For instance, studies illustrate that a polymer’s molecular weight and sterilization procedure affect biological and microstructural attributes of the scaffold [179,180]. Therefore, in sync with the advances in polymer modification approaches, investigations must equally focus on the way these modifications affect the architecture and biological performance. This would enable the development of preparations compatible with regulatory standards worldwide and those showing a lesser rejection in clinical settings. Results on larger patient cohorts will indeed show the footprints of scaffold research on clinical medicine in the future.

## Figures and Tables

**Table 1:** Summary of important parameters for tissue scaffold development.

General Attributes	Biocompatibility	Biological Signaling
Composition and porosity	Predictable degradation	Mimicry to the native environment
Stiffness and elasticity	Low immunogenicity	Release of cooperative factors
Formulation development and payload incorporation	Non-toxic degradation products	Colonization of host cells without inducing any histological changes
Ease of administration	Payload release	Integration with host tissues

**Table 2:** Polysaccharide based tissue scaffolds.

Components	Formulation	Application	Suggested Merits	Reference
CHI, PCL and polypyrrole	Electrospun nanofibres	neural tissue substitute	enhanced attachment and proliferation of PC12 cells	[20]
GEL and carboxymethyl CHI	Lyophilization	dermal tissue engineering	adhesion, growth, and proliferation of 3T3 mouse fibroblasts	[22]
maleiated CHI and thiol-terminated PVA	photocrosslinkable hydrogel	engineering of chondrocytes	rapid gelation, improved mechanical properties, and higher proliferation of L929 cells	[68]
CHI and COL	solvent casting	hepatocyte attachment	fetal porcine hepatocytes survived at least 14 days	[69]
ALG and some surfactants	Lyophilization	delivery of mesenchymal stem cells	sustained mesenchymal stem cell proliferation up to 14 days and improved release of growth factors	[14]
ALG	Lyophilization	soft tissue repair	differentiation of adipose-derived stem cells into adipocytes along with angiogenic action	[5]
ALG and SWCNTs	multinozzle deposition of the components	proliferation of endothelial cells	improved adhesion and proliferation of rat heart endothelial cells due to incorporated SWCNTs	[19]
Quaternized CHI polyaniline and oxidized DEX	lyophilized hydrogel	in situ forming antibacterial and electroactive hydrogels	high antibacterial activity and enhanced proliferation of C2C12 myoblasts in comparison to quarternized CHI hydrogel	[23]
PUL-DEX	Lyophilization	adherent cell growth	zero-order release of BSA and VEGF	[70]
RGD peptide functionalized DEX	crosslinked hydrogel	cell-homing scaffold	0.1% of RGD-modified DEX was sufficient to support HUVEC cells adhesion	[24]
Maleiated HA/thiol-terminated PEG	mould-casting	in-situ formable scaffolds	quick gelation, porous structures, tunable degradation, and cytocompatibility with L929 cells	[71]
CHI, HA and andrographolide	Lyophilization	wound care scaffold	enhanced wound healing and improved tissue quality	[72]
Thiophene ethylamine modified HA	Lyophilization	hepatocytes culture	improved expression of hepatic functional genes in primary mouse hepatocytes	[73]
Thiolated HA	Lyophilization	culture of fibroblasts and chondrocytes	improved density of living cells during culture for 28 days in vivo	[25]
HA and COL	Lyophilization	brain tissue engineering	improved mechanical properties through complexation of HA with COL	[74]
HA, GEL and CS	Lyophilization	retinal regeneration	favored differentiation of stem cells into retinal cell types and elicited a minimal immune response in mouse	[75]
DEX and PLGA	electrospinning	fibroblast/ macrophage co-culture	synergistic coordination of macrophages and fibroblasts stimulated the degradation rate scaffolds in comparison to counterparts incubated with a single type of cells	[76]
DEX and CHI	solvent casting	wound healing	deposition of ordered collagen and fibroblast migration	[77]

**Abbreviations:** Poly(ε-caprolactone), PCL; chitosan, CHI; gelatin, GEL; xanthan gum, XG; collagen, COL; alginate, ALG; pullulan, PUL; dextran, DEX; chondroitin sulphate, CS; poly(lactic acid-co-glycolic acid), PLGA; basic fibroblast growth factor, bFGF; bovine serum albumin, BSA; polyvinyl alcohol, PVA; matrix metalloproteinase, MMP; single-walled carbon nanotubes, SWCNT; arginine-glycine-aspartate, RGD; poly (ethylene glycol), PEG; vascular endothelial growth factor, VEGF.

**Table 3:** Summary of human clinical studies exploring the efficacy of polysaccharide scaffolds.

Scaffold Composition	Application	Study Design	Major Findings	Reference
Calcium-ALG hydrogel composed of Na^+^-ALG and Ca^2+^-ALG suspended in 4.6% aqueous mannitol	improvement of cardiac function in patients with heart failure	11 patients (males, age 44 to 74) with symptomatic heart failure; New York Heart Association class III or IV	scaffold placement along with coronary artery bypass grafting successfully induced remodeling and local stress reduction in the myocardial wall	[162]
	improvement of exercise capacity and symptoms in chronic heart failure	multi-centre, prospective, randomized trial involving 40 patients, 63 ± 10 years	ALG-hydrogel in addition to standard medical therapy was more effective in advanced chronic heart failure	[26]
1% ALG and 0.3% calcium gluconate (IK-5001)	reversal of left ventricular remodeling and dysfunction	27 patients (24 males, 03 females) with ST-segment–elevation myocardial infarctions; (mean age 54 ± 9 years)	provided initial proof on the tolerability of IK-5001 and the use of catheter-based strategy after myocardial infarction	[163]
ALG beads containing human mature allogenic chondrocytes	treatment of chondral lesions	21 patients (13 male, 8 female); mean age -33 years (12–47 years); mean lesion area-2.6 cm²; mean duration of symptoms-33.20 months (6–73 months)	clinical improvement in patients during 24 months of follow-up; histological analyses showed hyaline-like tissues (15.3%), mixed tissue (46.2%), fibrocartilage (30.8%), and fibrous (7.7%)	[15]
esterified HA seeded with autologous chondrocytes	knee cartilage defects	67 patients; mean follow-up time from implantation - 17.5 months	improvement in knee conditions (97%), quality of life (94%), surgeons’ knee functional test (87% of patients with the best scores), and cartilage repair (96.7% biologically acceptable)	[169]
	treatment of chondral knee lesion	16 patients (14 men, 2 women); mean age-31.5 years (range 16–42)	avoidance of open surgery, reduced surgical morbidity and operative time; functional capacity comparable to the standard techniques	[165]
	articular cartilage engineering	multicenter study on the cohort of 141 patients; follow-up time-2 to 5 years (average 38 months)	improvement in 91.5% of patients; 76% and 88% of patients had no pain and mobility problems; 95.7% patients showed normal knee with hyaline-like tissue	[168]
	treatment of full-thickness chondral defects	53 patients, mean age -32 ± 12 years, mean body mass index-24.5 ± 3.8kg/m^2^; mean defect size-4.4 ± 1.9 cm^2^	improvement of clinical outcome up to 7 years in healthy young patients with single cartilage defects; less complicated surgery and lower morbidity	[170]
			at a mean follow-up of 9.07 ± 2.9 years, treatment failure occurred in 22.6% cases at an average of 2.99 ± 1.40 years of surgery; significant clinical improvements	[167]
	hyaline cartilage regeneration	multicenter study 23 patients (18 men, 5 women), mean age-35.6 years, mean follow-up -16 months (range, 6–30); mean implant area-5.0 cm^2^	regeneration occurred in about 50% of patients during 6 to 30 month follow-up	[171]
